# Association of proprotein convertase subtilisin/kexin type 9 protein and oxidative stress indicators in women with preeclampsia: A case-control study

**DOI:** 10.18502/ijrm.v21i10.14540

**Published:** 2023-11-24

**Authors:** Anahita Abbasifard, Shohreh Alimohammadi, Ebrahim Abbasi Oshaghi, Gholamreza Shafiee

**Affiliations:** ^1^Department of Clinical Biochemistry, School of Medicine, Hamadan University of Medical Sciences, Hamadan, Iran.; ^2^Department of Obstetrics and Gynecology, School of Medicine, Hamadan University of Medical Sciences, Hamadan, Iran.; ^3^Department of Clinical Biochemistry, School of Medicine, Nutrition Health Research Center, Hamadan University of Medical Sciences, Hamadan, Iran.

**Keywords:** PCSK9, Hypercholesterolemia, Oxidative stress, Preeclampsia.

## Abstract

**Background:**

Proprotein convertase subtilisin/kexin type 9 (PCSK9) protein is one of the factors associated with oxidative stress and dyslipidemia disorders.

**Objective:**

This study aimed to evaluate the lipid profile, PCSK9 levels, and oxidative stress in preeclampsia.

**Materials and Methods:**

This case-control study was conducted at Sina hospital in Hamadan University of Medical Sciences, Hamadan, Iran from August 2020-May 2021. The average maternal age of included participants was 30 yr with 30 preeclampsia and 30 healthy pregnant women. After clinical examination, the fasting blood samples were collected, and the serum PCSK9 protein concentration, superoxide dismutase, glutathione peroxidase activities, and glutathione levels were determined by enzyme-linked immunosorbent assay. Total antioxidant capacity, total oxidant status, and malondialdehyde levels were determined manually.

**Results:**

The average maternal age of participants were 29.97 ± 4.75 and 31.23 ± 5.85 yr, respectively. The concentrations of total cholesterol, low-density lipoprotein cholesterol (LDL-C), PCSK9, total antioxidant capacity, and malondialdehyde levels were higher in the preeclampsia group compared with control (p < 0.02). Total oxidant status, glutathione levels, superoxide dismutase, glutathione peroxidase activities were lower in the cases group compared with the control group (p < 0.01). The PCSK9 variable had a significant negative association with antioxidant parameters; however, a significant positive association was observed between PCSK9 level and parameters of LDL-C.

**Conclusion:**

PCSK9 is associated with increased serum levels of LDL-C and oxidative factors in pregnant women that increase the risk of endothelial damage and hypertension in preeclampsia.

## 1. Introduction

Preeclampsia is characterized by high blood pressure, endothelial dysfunction, and vascular damage (1). Preeclampsia has an above prevalence of about 10% of pregnancies due to the mortality of pregnant women in many countries. Many hypotheses have been explained that the endothelial dysfunction, inflammation, and oxidative stress are the causes of preeclampsia (2).

Oxidative stress by metabolic changes causes vascular endothelial damage and hypertension, thereby increasing the risk of preeclampsia (3). Proprotein convertase subtilisin/kexin type 9 (PCSK9) is factor associated with oxidative stress. Accordingly, this protein is encoded in humans on chromosome 1 and is then expressed in many tissues and cells (4).

In addition, PCSK9 binds to the low-density lipoprotein cholesterol (LDL-C) receptor then the formed complex enters the cell and decomposes inside the lysosomes. Hence, it plays an important role in the regulation of cholesterol homeostasis. The decreased LDL receptor levels lead to decreased LDL-C metabolism, which can lead to hypercholesterolemia. Numerous studies consider elevated cholesterol, LDL-C, and more high-density lipoprotein cholesterol (HDL-C) levels as serious risk factors for preeclampsia (5).

Increased reactive oxygen species levels due to hypercholesterolemia also impair the protective function of antioxidant enzymes that include glutathione peroxidase (GPx), superoxide dismutase (SOD), and catalase, leading to an excessive increase in lipid peroxidation. An increase in oxidant compound finally improves the way of vascular endothelial dysfunction and increases the pathogenesis of preeclampsia (6).

Therefore, we aimed to investigate the relationship between lipid profile (triglyceride, cholesterol, HDL-C and LDL-C), PCSK9 concentration, and oxidative stress markers such as SOD, GPx, malondialdehyde, glutathione, total antioxidant capacity, and total oxidant status levels in preeclampsia women.

## 2. Materials and Methods

In this case-control study, thirty healthy pregnant women and thirty preeclampsia women were selected after the clinical examination and approval by the gynecologist from August 2020-May 2021 (Hamadan-Iran). The criteria of blood pressure ≥ 140/90 mmHg and proteinuria ≥ 300 mg/24 hr after 20 ${}^{\text{th}}$ wk of gestational age were included and diagnosed as preeclampsia.

The individuals of groups were matched according to the age, gestational age, and body mass index (BMI). Women with diabetes, thyroid, kidney, liver, respiratory problems, infections, heart disease, and smoking were excluded from the study. Blood samples were collected, then serum was separated and stored at -20 C. Blood pressure, weight, and height were determined. Demographic characteristics including age, gestational age, and used drugs, were obtained by a general information questionnaire.

### Lipid profile 

Fasting blood samples were collected, and the serum was separated. The plasma total cholesterol (TC), triglycerides (TG), HDL-C, and LDL-C concentrations were measured by an automated analyzer (Hitachi, Japan) and calculated using the following formula: LDL = TC - HDL - 0.2 × TG.

### PCSK9 protein 

Human serum PCSK9 concentration was assayed by using of enzyme-linked immunosorbent assay Kit (ZellBio GmbH, Germany) by a reader (synergy HTX, Biotech, USA). The standard PCSK9 range is 12.5-200 ng/ml.

### Total antioxidant capacity (TAC)

TAC was detected using the ferric-reducing ability of plasma manual method. The copper ion forms a dye and is measured by a spectrophotometer (Bell-Italy) at 532 nm. In this method, using the standard curve, the concentration of samples were determined (7). For calculation of the TAC level, a standard curve was designed, and then the regression equation y = mx ± b was used.

### Total oxidant status (TOS)

Total oxidative status was assayed manually. The ferrous ion is oxidized to the ferric, and this ferric ion complex with the giselle orange with colored complex in acidic conditions. It was measured using a spectrophotometer (Bell-Italy) at 532 nm (7). For calculation of TOS, a standard curve was designed, then the regression equation y = mx ± b was used.

### Malondialdehyde (MDA)

An amount of 500 µL of serum was added to 2500 µL of trichloroacetic acid (20%), then incubated at room temperature for 10 min and then centrifuged for 10 min at 3000 g, and the precipitate was washed with sulfuric acid. The sulfuric acid and thiobarbituric acid (0.2%) solution were added to the precipitate, and the samples were incubated in the standard solution (0.5, 1, 2, 4, 8, 12, and 20 μM tetraoxypropane in 5% sulfuric acid) boiling at 100 C for 30 min. Next, n-butanol was added to tubes and centrifuged at 3500 g for 10 min. The solution absorbance was measured at 532 nm (8).

### Growth hormone (GH) 

GH concentration was assayed using an ELISA Kit (Cat. ZB-GSH-96A, Ulm, Germany). That glutathione was reacted with 5, 5 ' -dithiobis-2-nitrobenzoic acid, and the maximum absorbance of the yellow 2-nitro 5-thiobenzoic acid was measured at 412 nm (9).

### SOD enzyme activity

Serum super oxidase dismutase activity was determined using the ZellBio Kit (ZellBio GmbH, Germany). The superoxide anions are changed to hydrogen peroxide and oxygen under enzymatic reaction. Finally, the products produce chromogenic products that can be read in terms of calorific value at 420 nm and data reported as U/mg protein. SOD was calculated by following the formula:

 Inhibitory percentage = (A ${}_{B1}$ -A ${}_{B2}$ )-(A ${}_{T}$ -A ${}_{C}$ )/(A ${}_{B1}$ -A ${}_{B2}$ ) × 100

The absorbance for control, standard test, and blank were recorded as AT, AC, AS, and AB, respectively.

### GPx enzyme activity

GPx activity was determined by the colorimetric kit (ZellBio GmbH, Germany). The enzyme changed glutathione (GSH) to Glutathione disulfide, and GSH reacted with 5, 5 ' -dithiobis-2-nitrobenzoic acid and produced a yellow color that was absorbed in 412 nm. Finally, the GPx activity was reported as U/mg protein. GPx was calculated by the following formula:

Inhibitory percentage = (A ${}_{C}$ -A ${}_{T}$ )/(A ${}_{C}$ -A ${}_{B}$ ) × 100

The absorbance for control, standard test, and blank is recorded as AT, AC, AS, and AB, respectively.

### Ethical considerations

This case-control study was approved by the Ethics Committee of Hamadan University of Medical Sciences, Hamadan, Iran (Code: IR.UMSHA.REC.1398.570). The informed consent was obtained from all the participants.

### Statistical analysis

Data were analyzed using the statistical software SPSS 16 (SPSS Inc. Chicago, USA). Pearson regression analysis and association test were used to investigate the relationship between the variables. Values are given as mean ± SD. The significance level of all statistical tests was < 0.05.

## 3. Results

### Demographic characteristics findings

From a total of 60 women, the average maternal age of participants was 29.97 ± 4.75 and 31.23 ± 5.85 yr, respectively, with no statistical difference between groups. Also, no statistical differences were observed in BMI, gestational age at sampling, and systolic blood pressure between the 2 groups; all individuals were above the 20 ${}^{\text{th}}$ wk. The results showed a significant difference in gestational age at delivery, neonatal weight, diastolic blood pressure, and proteinuria between 2 groups (Table I).

According to the findings, the concentrations of TC and LDL-C were higher in the preeclampsia group than that in the control group. No statistical difference was observed in TG and HDL-C between groups (Table II).

### PCSK9 protein

**Figure 1:**
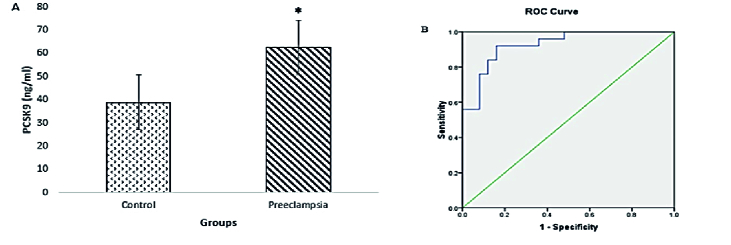
(A) The concentration of PCSK9 protein in the preeclampsia group compared to the control group shows a significant increase (p < 0.001). The symbol (*) represents significant differences (p < 0.05). (B) The results of the analysis of the ROC diagram in PCSK9 factor for preeclampsia [0.913 (95% CI, 0.88-0.975)].

PCSK9 concentration in the preeclampsia group showed a significant increase (p < 0.001) compared to the control group (Figure 1A). Receiver operating characteristic curve (ROC) analysis was performed to investigate whether the PCSK9 protein factor could be considered a diagnostic factor for preeclampsia. The analysis results below the ROC diagram in the PCSK9 factor for preeclampsia cases and control group were obtained as [0.913 (95% CI, 0.88-0.975)] (Figure 1B). The PCSK9 concentrations in the control and cases groups are presented in table II.

### TAC and TOS 

The finding showed a significant decrease in the patient's TAC index compared to the control group (p = 0.01). Comparison of the mean TOS factor in serum between different groups showed that the amount of TOS in the patient group increased significantly compared to the control group (p = 0.03) (Table II).

### MDA 

The MDA level in the preeclampsia group and normal pregnancy was 6.55 and 3.82 µmol/L, respectively. The MDA level in the 2 groups, preeclampsia and control, was statistically significant (p = 0.03) (Table II).

### Glutathione 

The serum glutathione had a significantly lower level in preeclampsia compared to the control group (p = 0.01), as shown in table II.

### SOD and GPx activity

The results of SOD enzyme activity showed that the activity of this enzyme was not significantly different in the patient group compared to the control group (p = 0.05). The results of GPx enzyme activity are given in similar results. As can be seen, a significant reduction was observed in the amount of this enzyme in the cases group compared to the control group (p = 0.02) (Table II).

### Association analysis of serum PCSK9 with LDL-C, SOD, GPx, TOS and TAC levels 

According to association analysis, the PCSK9 in the control group had significantly negative association with SOD (p = 0.03, β = -0.638), GPx (p = 0.04, β = -0.002), GSH (p = 0.03, β = -0.238), and TAC (p = 0.02, β = -0.466). But in this group, a significant positive association was seen between PCSK9 level and factors of LDL-C (p = 0.01, β = 0.709), MDA (p = 0.02, β = 0.676), and TOS (p = 0.01, β = 0.473). Also, the PCSK9 in the preeclampsia group had a significant negative association with SOD (p = 0.07, β = -0.596), GPx (p = 0.04, β = -0.015), GSH (p = 0.01, β = -0.426) and TAC (p = 0.04, β = -0.585). But in this group, there was a significant positive association between PCSK9 level and 2 parameters of LDL-C (p = 0.01, β = 0.734), MDA (p = 0.03, β = 0.671), and TOS (p = 0.04, β = 0.399).

**Table 1:** Demographic parameters in control and preeclampsia groups (n = 30/each)


**Variable**	**Control group**	**Preeclampsia group**	**P-value**
**Gestational age at sampling (wk)**	31.12 ± 1.07	30.65 ± 2.67	0.422
**BMI at sampling (kg/m^2^)**	23.62 ± 4.71	24.61 ± 3.15	0.223
**Maternal age (yr)**	29.97 ± 4.75	31.23 ± 5.85	0.150
**Systolic blood pressure (mmHg)**	118.89 ± 8.28	150.40 ± 13.94	0.064
**Diastolic blood pressure (mmHg)**	72.64 ± 6.18	94.83 ± 10.28	0.001
**Proteinuria (mg/24 hr)**	142.72 ± 6.43	305.14 ± 13.51	0.001
**Gestational age at delivery (wk)**	39.27 ± 1.05	35.43 ± 1.25	0.032
**Neonatal weight (kg)**	3.48 ± 0.72	2.56 ± 0.36	0.001
Data presented as Mean ± SD. *t* test. BMI: Body mass index			

**Table 2:** Comparison of baseline lipid profile and TAC, TOS, MDA, GSH, GPx, and SOD levels between groups (n = 30/each)


**Variables**	**Control**	**Preeclampsia**	**P-value**
**TG (mg/dl)**	197.36 ± 6.72	208.29 ± 9.89	0.312
**TC (mg/dl)**	172.20 ± 11.51	231.75 ± 12.53	0.026
**HDL-C (mg/dl)**	63.85 ± 5.71	59.14 ± 4.16	0.148
**LDL-C (mg/dl)**	107.73 ± 10.35	153.57 ± 12.45	0.019
**TAC (µmol/ml)**	2442.80 ± 175.24	1911.29 ± 276.43	0.015
**TOS (µmol/ml)**	12.67 ± 9.68	19.17 ± 11.89	0.028
**MDA (µmol/L)**	3.55 ± 0.11	6.82 ± 0.09	0.032
**GSH (µmol/L)**	163.61 ± 9.24	98.17 ± 5.17	0.012
**SOD (IU/ml)**	26.21 ± 3.47	14.59 ± 2.18	0.028
**GPX (IU/ml)**	79.38 ± 6.56	43.06 ± 3.43	0.019
**PCSK9 (ng/ml)**	35.90 ± 6.02	65.62 ± 5.87	0.023
Data presented as Mean ± SD. *t* test. TG: Triglyceride, TC: Total cholesterol, HDL-C: High-density lipoprotein cholesterol, LDL-C: Low-density lipoprotein cholesterol, TAC: Total antioxidant capacity, TOS: Total oxidative status, MDA: Malondialdehyde, GSH: Glutathione, SOD: Superoxide dismutase, GPX: Glutathione peroxidase, PCSK9: Proprotein convertase subtilisin/kexin type 9			

## 4. Discussion

In this study, we examined the levels of PCSK9 protein factor, as an indicator of oxidative stress, in women with preeclampsia. Also, oxidative stress factors such as TAC, TOS, MDA, GSH, and the activity of SOD and GPx antioxidant enzymes were measured in this study. By investigating the serum concentration of PCSK9 factor in the patients with preeclampsia and comparing it with the control group, a significant increase in this factor was observed in the patient group compared to the control group.

In the present study, according to the many studies the increased of PCSK9 due to the increased LDL oxide and oxidative stress (10). PCSK9 protein is an important mediator in LDL-C control and plays a role in mediating LDL receptor degradation (11). Moreover, PCSK9 is expressed in many tissues such as the liver, small intestine, kidneys, and hippocampus and cerebellum of brain tissue. In addition, many studies have shown the importance of the MAPK pathway in PCSK9 expression (12). The phosphorylation and activity of the p38 mitogen-activated protein kinase factor, which is the location of the source cell, increases the activity and expression of the hepatocyte nuclear factor 1α in the nucleus, which subsequently increases the hepatocyte nuclear factor 1α transcription and expression of the *PCSK9* gene. Many studies have shown that mutations in the *PCSK9* gene led to some diseases, such as hypercholesterolemia and atherosclerosis (13). Similar to previous studies, increasing the serum level of PCSK9 due to increased LDL-oxide levels and the expression of low-density lipoprotein receptor-1, which in the form of pathways can increase PCSK9 expression at later stages (14).

The finding of our study showed that TC and LDL-C concentrations were higher in the preeclampsia group. Previous studies showed that preeclampsia is associated with enhanced hyperlipidemia, which seems to have a negative effect on fetal (15).

It has been revealed that LDL-C and high oxidized LDL increase oxidative stress and play a causal role in preeclampsia. Oxidized LDL itself acts as a factor in the induction of increasing oxidant factors (TOS and MDA) compared with antioxidants (such as TAC, GSH, SOD, and GPx) (16). Many biological effects of OxLDL is the generation of more cellular ROS by several pathways. So, it exists an imbalance of oxidant and antioxidant systems. In the present study, a significant decrease was observed in the TAC of the preeclampsia group compared to the control group. However, the TOS index was significantly higher in the patient group. Inconsistent with our findings, it was shown that hypercholesterolemia leads to excessive lipid peroxidation and imbalance between prooxidants and antioxidants and may contribute to atherogenicity in preeclampsia (17).

A significant decrease in TAC levels and a significant increase in TOS and MDA levels were reported by measuring the redox status in the serum of the major depressive disorder patients and then compared with the control group. A study reported no significant change in serum antioxidant capacity in patients with neurological diseases compared with the healthy pregnant control group. However, a decrease in serum TAC in people with preeclampsia was insufficient to protect the environment against free radical attack. In this study, the activity of GPx and SOD enzymes was significantly lower in the serum of the patient group compared to the control group. In addition, the concentration of TAC and GSH in the patient group was significantly lower than in the control group (18).

In line with previous studies, decreased activities of SOD and GPx enzymes and GSH levels were found in the serum of preeclampsia patients compared to the control group. Moreover, the association oxidative stress with preeclampsia has been shown in several studies (18). Other studies showed that the activation of oxidative stress pathways in preeclampsia was associated with the increase of the inflammatory factors by the phosphatidylinositol-3 kinase/Akt/mammalian target of rapamycin pathway activation. Therefore, changes in serum antioxidants among preeclampsia could be considered another reason for their possible role in preeclampsia. So, PCSK9 can be an important factor in increasing oxidative stress. In a study, by using PCSK9 siRNA, the effect of the PSCK9 factor on oxidative stress was detected by the nuclear factor kappa B pathway (19).

Finally, according to the findings, the relationship between PCSK9 and LDL-C variables, similar to MDA and TOS, is significantly positive in the 2 groups, which confirms our study thesis. This means that the PCSK9 protein has a direct relationship with LDL-C as an oxidative factor and due to increase in MDA and oxidative stress. Therefore, it can be claimed that PCSK9 was considered an important factor in the changes of oxidative stress and hypertension in preeclampsia. The results of this study show a high level of PCSK9 and oxidative stress levels as well as its relationship with preeclampsia. In general, the results of our study confirmed the role of PCSK9 and oxidative stress and its related indicators in preeclampsia. So, it may be said that PCSK9 protein can be involved in oxidative stress and was affected through certain pathways in the occurrence of preeclampsia.

##  Conflicts of Interest

The authors declare that there is no conflict of interest.
